# Supplementary material for: Neurodevelopmental problems and extremes in BMI
Source: PeerJ. 2015 Jul 7;3:e1024. doi: 10.7717/peerj.1024 (PMC4511820; doi:10.7717/peerj.1024)
Supplement: Appendix S1 [file peerj-03-1024-s001.docx]

**Appendix. Supplementary Information**

***Box-Cox transformation***

This method belongs to the family of power transformations capable of improving the normality of a distribution of a chosen variable. Data transformations are normally used for data intended for analyses with parametric tests (e.g. ANOVA and *t*-test) that do not fulfill the required assumptions of either normal distribution (e.g., when distribution is skewed) or homogeneity of variance ([Osberne 2010](#_ENREF_2)). The raw BMI data distribution did not follow the hypothesized normal distribution, but was positively skewed.

Box-Cox transformation defined as y = (xλ-1)/λ when λ ≠ 0 ([Box & Cox 1964](#_ENREF_1)) differentiates itself among conventional transformation techniques such as square root and log transformation by its ability to optimize the transformation for the chosen variable ([Osberne 2010](#_ENREF_2)). This optimization is accomplished by the potential of Box-Cox to vary the range of λ ([Osberne 2010](#_ENREF_2)). Ideal settings for a normally distributed curve should have skewness close to 0.00 and range between −0.8 and +0.8 ([Osberne 2010](#_ENREF_2)). Kurtosis should be around 3 for normal distribution, but is defined as 0 in SPSS ([Osberne 2010](#_ENREF_2)).

In the present study we conducted 4 transformations as described below.

First we empirically estimated λ for each subgroup (9-year-olds/12-year-olds, boys/girls) according to the description published by Osborne in 2010. By transforming each subgroup simultaneously, with the starting range for λ of −1.5– 0 and with intervals of 0.05 between each transformation, these transformations resulted in a total of 31 histograms with transformed BMI values for each subgroup. A table of skewness revealed which of these transformations (each with its unique λ-value) exhibited the smallest amount of skewness. By repeating this process for each subgroup, an optimal λ for specific subgroups could be found.

Descriptive data derived from the Box-Cox transformation

|  | | | | | | | |
| --- | --- | --- | --- | --- | --- | --- | --- |
| Population | | Box-Cox | | | Correction | | Mean BMI |
| Gender &  age | n | λ | Transformed mean | Transformed SD | Skewness | Kurtosis |  |
| **9-year-old boys** | 2819 | −1.10 | 0.867 | 0.00577 | 0.010 | 0.459 | 0.0000 |
| **12-year-old boys** | 3077 | −1.25 | 0.778 | 0.00372 | -0.009 | 0.159 | 0.0000 |
| **9-year-old girls** | 2585 | −0.85 | 1.066 | 0.01285 | -0.006 | 0.452 | 0.0000 |
| **12-year-old girls** | 2874 | −0.90 | 1.027 | 0.01087 | 0.007 | 0.572 | 0.0000 |
| BMI = body mass index  SD = standard deviation | | | | | | | |

**References**

Box JEP, Cox DR. 1964. An analysis of transformations. *Journal of the Royal Statistical Society* 26:211-234.

Osberne J. 2010. Improving your data transformations: applying the Box-Cox transformation. *Practical Assessment, Research & Evaluation* 15:1-9.
